# Assessing timewise changes over 15 months in life-space mobility among community-dwelling elderly persons

**DOI:** 10.1186/s12877-020-01882-4

**Published:** 2020-11-25

**Authors:** Chisato Hayashi, Haruka Tanaka, Soshiro Ogata

**Affiliations:** 1 University of Hyogo, 13-71 Kitaoji-cho, Akashi, Hyogo 673-8588 Japan; 2 Nagoya University, Graduate School of Medicine, 1-1-20 Daiko-Minami, Higashi-ku, Nahgoya City, Aichi Prefecture 461-8673 Japan; 3 Department of Preventive Medicine and Epidemiology, National Cerebral and Cardiovascular Center, 6-1, Kishibe - Shimmachi, Suita, Osaka, 564-8565 Japan

**Keywords:** Life-space mobility, Living alone, Social frailty, Multilevel analysis

## Abstract

**Background:**

The purpose of this study was to examine the differences of timewise changes in life-space mobility between elderly people living alone and those living with others among community-dwelling elderly people from a day care facility with a rehabilitation service for seniors.

**Methods:**

The present study used a longitudinal design with repeated measures every 3 months. In conformity with our inclusion criteria, this study included 233 community-dwelling elderly users of a day care facility with rehabilitation services for seniors in Japan. We analyzed the life-space assessment (LSA) scores collected at five time points (baseline, 3 months, 6 months, 9 months, and 12 months) using mixed-effects models with random intercepts and slopes over time. In the present study, the explanatory variables of interest were time, and living situation (living alone or with others). As possible confounders, we considered the following: (a) age, (b) sex, (c) social frailty, (d) physical frailty, (e) mild cognitive impairment (MCI), (f) depression, and (g) economic satisfaction.

**Results:**

The mean age of participants was 78.9 years (SD = 7.7), their mean LSA score was 60.1 points (SD = 25.7), and 42.9% of the participants were men. After adjusting for age, gender, frailty, depression, MCI, and economic satisfaction, the mean LSA score of older adults who lived with others was significantly lower (7.42 points, 95%CI = − 18.30 to − 0.15, *p* = 0.048) than that older adults who lived alone.

**Discussion:**

Community-dwelling older adults who used a day care center with rehabilitation services and lived with others had a smaller life-space at baseline than those who lived alone. This suggests that there is a need to pay more attention to social frailty among both older adults who live alone and those who live with others.

**Conclusions:**

According to a multilevel analysis growth model, elderly persons who lived with others had significantly lower life-space mobility than those who lived alone.

**Supplementary Information:**

The online version contains supplementary material available at 10.1186/s12877-020-01882-4.

## Background

In Asian and western countries, many studies have been conducted with elderly individuals who live alone [1–3]. There is evidence that living alone is associated with poor health, multiple falls, functional impairment, risk of social isolation, and chronic conditions like arthritis and/or rheumatism, glaucoma, and cataract [4]. However, there is also some evidence that suggests that the elderly individuals living alone should not be regarded as a population with many special problems [5]. Moreover, the World Health Organization has reported that elderly people living alone are an at-risk group; however, research in many cultural settings shows that older people prefer to live alone in their own homes [6].

In Japan, research on the houseboundedness of elderly persons living alone has increased since around 2000, and being housebound was incorporated as one of the risk factors when the nursing care prevention system was implemented in 2006 [7]. Since then, houseboundedness has been identified as a risk factor for nursing care needs by a comprehensive care prevention checklist. Extent of daily activity, frequency of going out, frequency of contact with others, and mobility were reported as four elements that characterize Japanese housebound individuals [7].

Age-related decline in life-space mobility is observed even in the absence of disease; however, some elderly adults experience abnormal declines in life-space mobility over short periods. Life-space mobility may be one of the most important means by which to increase well-being in community-dwelling elderly persons [8]. Life-space mobility is a holistic measure of resilience to physical and social isolation in late life [9]. There is evidence that a relationship exists between psychosocial factors, such as race [10] and sex [11], and life-space mobility. In an elderly Japanese community, life-space assessment via path analysis suggested that age, skeletal muscle mass, fear of falling, and mobility had direct effects on life-space mobility, whereas lower extremity muscle strength and cognitive function affected life-space mobility indirectly [12].

Over recent decades, frailty has received increasing worldwide attention from researchers. Recent research has assessed the relationship between frailty and risk factors for falls [13], hospital use and mortality [14], sarcopenia and osteoporosis [15], dental care [16], oral environment [17], and mental and physical comorbidity [18]. Although there is no consensus for definition of frailty, the two most common measurements of frailty are Fried’s Frailty Phenotype [19] and the Frailty Index (FI) [20]. The concept of houseboundedness is not considered an aspect of the frailty framework by Gobbens et al. [21]; however, for a long time, it was included in the frailty checklist adopted by the nursing care prevention system in Japan [22].

A recent study reported that social frailty might precede and lead to the development of physical frailty [23]. A review of 42 studies of social frailty defined it as a continuum of being at risk of losing, or having lost, resources that are important for fulfilling one or more basic social needs during an individual’s life span [24]. Social frailty is the most unexplored aspect of the overall concept of frailty, which includes physical, psychological, and social components. Moreover, there is no consensus regarding which indexes should be used to screen for social frailty [25, 26].

There are four diagnostic tools for social frailty relevant to long-term care needs in Japan. First, there is the social frailty screening index, which is based on the concept of social frailty developed by Bunt et al. [24]. Second, there is the social frailty index, which is a measure of social frailty created in a Singapore cohort study [25]. Third, there is the National Center for Geriatrics and Gerontology - Study of Geriatric Syndromes (NCGG-SGS) Social Frailty Diagnostic Criteria [27]. Lastly, a comprehensive index of frailty, included in the Kaigo-Yobo checklist (KYCL), is being used in Japan to assess social frailty [28]. The KYCL is a comprehensive frailty index; however, it also incorporates a framework based on the concept of houseboundedness within the Japanese context, which has been shown to be highly correlated with the FI [23].

To our knowledge, Que. et al. [29] published the first study focused on the relationship between frailty and life-space mobility and found that a slightly constricted life space may be a marker and/or risk factor for the development of frailty, which may prove useful as a screening tool or a target of intervention in community-dwelling women [29]. Another previous study found that sense of autonomy of physical performance explained up to 32% of the variation in the life-space mobility of elderly patients [30]. In addition, a cohort study reported that the age- and sex-adjusted generalized estimation equation model showed that life-space mobility was more limited among individuals in the pre-frailty and frailty groups, compared with those in the without-frailty group [31]. Although some studies on the relationship between life-space mobility and physical frailty exist, few have focused on the association between life-space mobility and social frailty among community-dwelling elderly people, including those who live alone.

The purpose of this study was to examine the differences of timewise change in life-space mobility between community-dwelling elderly adults living alone and those living with others who used a day care facility with rehabilitation services for seniors.

## Methods

### Study design and participants

We conducted a study of timewise changes among participants who used a day care center with a rehabilitation service for long-term preventive care in Hyogo, Japan. Individuals who expressed willingness to participate in the care-prevention campaign were recruited by the day care service for the study. We briefed all candidates in the day care center about the study and asked them if they would like to participate, after which we asked them to sign a consent form. This research was part of a care-prevention campaign in the day care center, and the intention of participation was respected by the individual and their family.

The inclusion criteria for participants in the present study were as follows: (a) male and female participants who took part in the longitudinal study every 3 months between August 2015 and April 2017, and (b) participant aged > 45 years. Participants with severe dementia and psychiatric disorders who had been diagnosed before beginning the present study were excluded based on information provided by their day care center, as they would have been unable to answer the questionnaire. Therefore, there were no participants diagnosed with dementia in the present study. In addition, when we went to the day care center to brief candidates about this study and to obtain their informed consent, we communicated with participants and written sign from them. All individuals who agreed to participate in this study filled an informed consent form. We collected information from 263 participants who met the aforementioned criteria. Out of 263 participants, 22 were excluded from the present analysis because they did not complete the LSA questionnaire, while and 8 were excluded because they did not answer the question regarding housemates, that is, whether they lived with others or not, as they could not be categorized into groups. Others were treated as missing values if the answers to the questions were unclear.

### Measurements of the outcome variable

The present study assessed changes in life-space mobility among community-dwelling elderly individuals over 12 months. We measured life-space mobility using the Aging Life-Space Assessment (LSA) tool, which measures mobility based on the distance a person reports having moved over the previous 4 weeks [32]. The LSA is particularly useful to evaluate mobility in community-dwelling older adults because it consists of only 15 items that can be used in face-to-face and telephone interviews. In addition, while other scales measuring level of disability are often subject to floor or ceiling effects, the LSA measures the full continuum of mobility among community-dwelling older adults [33]. The life-space score is stable over a two-week period, yet it is sensitive to changes over 6 months [32].

The LSA measures a person’s mobility over a four-week period. The assessment consists of questions with respect to five spaces: bedroom (level 0), other rooms besides the bedroom (level 1), outside the home (level 2), neighborhood (level 3), within the town (level 4), and within other towns (level 5). The LSA measures the frequency with which the individual visits each space: less than once per week (score 1), 1–3 times per week (score 2), 4–6 times per week (score 3), or daily (score 4). The LSA also measures independence, namely, whether the respondent needed help from another person or whether they used aids or equipment (1 = personal assistance, 1.5 = equipment only, and 2 = no equipment or personal assistance). A composite score is calculated by summing the level score (from 1 to 5), frequency score (from 1 to 4), and assistance score (1, 1.5 or 2). The total score ranges from 0 to 120, where higher scores indicate greater life-space mobility.

### Measurements of explanatory variables

In the present study, the explanatory variables of interest were time and living situation (living alone or with others). Time was based on timing of the LSA measurement at baseline, 3 months, 6 months, 9 months, and 12 months. The regression coefficient of time represented LSA score’s slope per 3 months (i.e., mean differences in LSA score in relation to time changes). We asked the question, “Are you living alone? Yes or No.” If they answered “no,” we asked them to elaborate on their family structure.

### Measurements of possible confounders

As possible confounders, we considered the following: (a) age, (b) sex, (c) social frailty, (d) physical frailty, (e) mild cognitive impairment (MCI), (f) depression, and (g) economic satisfaction. Age was used for analysis by centering on the mean age of the entire group (78.9 years). Therefore, the intercept of the LSA estimate showed a value of 78.9 years old.

Social frailty was assessed using five questions regarding houseboundedness from the Kaigo-Yobo Checklist (KYCL) [28]. Physical frailty was assessed using six questions that obtained data regarding falling and four questions about nutrition from the KYCL. The total score ranges from 0 to 15, with a higher score indicative of greater frailty [28]. This checklist consists of 15 easy-to-answer questions in three main categories: houseboundedness, falling, and low nutrition. The houseboundedness category consist of five items: *Do you spend most of your time inside your house instead of being outside all day?, Do you have any hobbies that bring you joy?, Do you have any neighbors you enjoy talking to?, Do you have anyone that you visit regularly or who visits you?, and How frequently do you go out for work, including farm work, shopping, walks, and hospital visits?.* The Falling category consists of six items: *Did you have history of falling within the past year?, Do you have a fear of falling?, Do you often trip or slip in your house?, and Can you walk continuously for about 1 km?, and Do you have low vision?.* The poor nutrition category includes the following items: *Have you been hospitalized within the past year?, Do you have good or poor appetite?, What amount of food can you chew?, Have you lost weight (3 kg or more) over a six-month period?, and Have you lost muscle mass or fat at an accelerated rate over the last 6 months?.* This scale has a sensitivity of 0.70 and a specificity of 0.89, and uses Fried’s frailty framework as an external criterion. It has also been validated by using activities of daily living (ADL), disability, and the level of care required as outcomes. Therefore, it is considered a simple scale that can be used as an index of frailty in Japan. Individuals with scores of four or more points were determined to exhibit frailty.

Cognitive function was measured using the Japanese version of the Montreal Cognitive Assessment instrument (MoCA-J) [34]. The MoCA assesses nine domains of cognition: attention, concentration, executive function, memory, language, visual construction skills, conceptual thinking, calculations, and orientation. Cronbach’s alpha for the MoCA-J was 0.74 when originally developed [34]. Using a cut-off score of 25/26 (total score range: 0–30), the MoCA-J demonstrated a sensitivity of 93.0% and a specificity of 87.0% for screening mild cognitive impairment (MCI). A recent study reported that across all subjects, the mean MoCA-J score was 20.1 ± 4.6 in Japanese community-dwelling elderly adults. As a result, 44 of the 48 subjects (92%) had a cut-off value of 25 points or less, indicating mild cognitive decline [35]. Another study reported that MCI screening in the community identified that more than 60% of older adults had MCI [36]. The MoCA-J faces some challenges but it is superior in longitudinal tracking, enabling early detection of high-risk groups in the region [36]. Additionally, previous research has reported that mobility declines could be features of MCI [37, 38].

Additionally, depression was assessed using the Geriatric Depression Scale-15 (GDS-15) and economic satisfaction was assessed with the following question: *How satisfied are you with your financial situation (very satisfied, sufficiently satisfied, neutral, somewhat satisfied, and not sufficiently satisfied).*

### Ethics approval

This study received approval from the Ethics Committee of the Fujita Health University (No. HM19–244).

### Statistical analyses

We analyzed the LSA scores collected at five time points (baseline, 3 months, 6 months, 9 months, and 12 months) using mixed-effects models with random intercepts and slopes over time. We modeled as fixed effects living situation (living alone or with others), age, gender, frailty, and time, while we controlled for MCI, depression, economic satisfaction, and an interaction between time and living situation. Time was coded as 0, 1, 2, 3, and 4, which corresponding to the baseline, 3 months, 6 months, 9 months, and 12 months, respectively, so as to ensure that the time regression coefficient represented change in LSA score per 3 months. We investigated associations between living situation, and changes in LSA scores via four models. In Model 1, the association was adjusted for age, gender, and time. Model 2 was based on Model 1, with additional adjustment for economic satisfaction, depression, and MCI. Model 3 was adjusted for frailty but each question was put into the model. Lastly, Model 4 was based on Model 3, with additional adjustment for economic satisfaction, depression, and MCI. From the models, we reported regression coefficients, 95% confidence interval (95% CI), and *P* values. We also examined model residuals. Mixed-effects models can adequately handle missing values of the outcome variable, with a missing-at-random (MAR) assumption. We used R version 3.6.3 software [39] for the analyses, with the lme4 package [40] to fit the mixed-effects models [41]. *Maximum likelihood methods were used for the analysis of missing data because the pattern of the missing data was not MCAR (missing completely at random).*

## Results

The comparison of baseline demographic characteristics is shown in Table 1. The participants’ mean age was 78.9 years (SD = 7.7), the mean life-space score was 60.1 points (SD = 25.7), and 42.9% of the participants were male. At baseline, 63.5% of participants were identified as frailty according to the Kaigo-Yobo Checklist (KYCL), 41.2% exhibited depression, and 68.8% were had MCI. The living alone group had a significantly higher LSA score than the living with others group. *The proportion of missing data for the life-space score was 34.4%, for those identified as frail was 1.2%, and for those with MCI was 4.6%*.

**Table 1:** Demographic comparison between groups (baseline)

	Total *N* = 233 mean ± SD	Living alone *N* = 75 mean ± SD	Living with others *N* = 158 mean ± SD	*p*-value
Age	78.9 ± 7.7	80.5 ± 7.9	78.0 ± 7.4	0.074
LSA	60.1 ± 25.7	67.0 ± 25.3	56.1 ± 25.5	< 0.05
-Male	55.6 ± 27.1	61.5 ± 31.0	54.0 ± 25.9	0.382
-Female	63.7 ± 24.1	69.2 ± 22.8	58.8 ± 24.9	< 0.05
Economic satisfaction	1.3 ± 1.1	1.2 ± 1.2	1.4 ± 1.1	0.340
	N(%)	N(%)	N(%)	
Male	100 (42.9%)	18 (18.0%)	82 (82.0%)	
Female	133 (57.1%)	57 (42.9%)	76 (57.1%)	< 0.001
Frailty +	146 (63.5%)	43 (29.5%)	103 (70.5%)	0.309
Depression +	96 (41.2%)	37 (38.5%)	59 (61.5%)	0.111
MCI+	155 (69.2%)	44 (28.4%)	111 (71.6%)	0.150

Changes in life-space score are shown in Table 2. The mean LSA total score was significantly higher among participants living alone versus those living with others at Time measure 1. Both groups showed improvements in LSA score, but the slope of improvement was better for older people with living others.

**Table 2:** Change in Life space score (Raw data)

Total						
score	Mean	SD	Median	Min	Max	
Baseline	60.1	25.7	59.8	9.0	120.0	
3 months	64.5	23.8	64.0	8.0	120.0	
6 months	68.2	25.4	67.5	12.0	120.0	
9 months	66.0	26.0	66.8	9.0	120.0	
12 months	64.5	26.3	65.5	16.0	120.0	
Living alone				Living with others		
score	Mean	SD		Mean	SD	p-value
Baseline	67.0	25.3		56.1	25.5	< 0.05
3 months	66.2	24.5		62.8	23.1	0.413
6 months	69.8	23.6		67.0	26.2	0.493
9 months	68.6	25.1		64.7	26.4	0.388
12 months	67.4	24.0		63.8	27.2	0.429

**Fig. 1:**
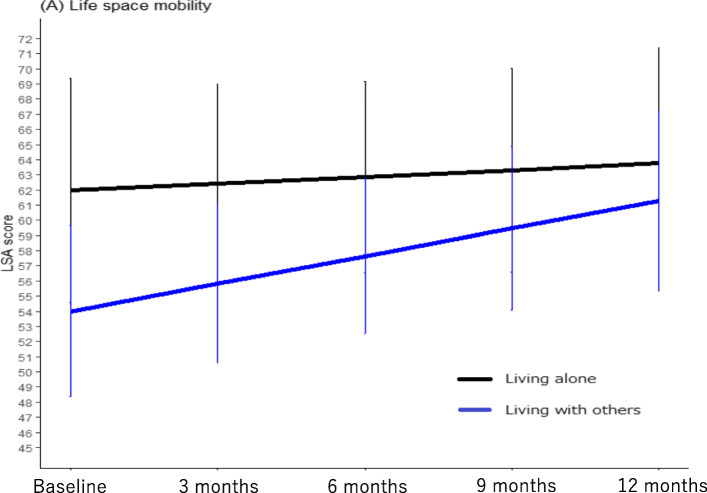
Slopes of changes and estimated marginal means (95% confidence interval) in the participants. The slopes and estimated marginal means were based on results described by Model2 in Table 3, which were obtained from linear mixed models. Bar lines show the 95% confidence intervals for the estimated marginal means for each time

Table 3 and Fig. 1 show regression coefficients obtained from the multilevel linear regression models that assessed the associations between risk factors at baseline and LSA scores at the five time points. The mean LSA score of older adults who lived with others was significantly lower (β = − 9.42, 95%CI = − 19.52 to − 2.96, *p* = 0.010) than that of older adults who lived alone in Model 1, after being adjusted for age, gender, and months from the baseline per 3 months. After adjusting for economic satisfaction, depression, and MCI in Model 2, the mean LSA score of older adults who lived with others was significantly lower (β = − 9.72, 95%CI = − 17.36 to − 2.11, *p* = 0.012) than that of older adults who lived alone. Intercept of the LSA score was 73.53 points (95%CI = 63.09 to 84.02, *p* < 0.001). The estimated marginal means are shown in Supplemental Table 1.

**Table 3:** The associations of living situation and life-space mobility

	Model1 Mean difference of LSA score by 1-unit increase of each explanatory variable (95%CI)		Model2 Mean difference of LSA score by 1-unit increase of each explanatory variable (95%CI)	
	66.75 (58.75, 74.90)	*P* < 0.001	73.53 (63.09, 84.02)	*P* < 0.001
**Age** (years old)	−0.51 (−0.87, − 0.15)	*P* = 0.006	−0.42 (− 0.82, − 0.02)	*P* = 0.038
**Female sex** (Men = Ref.)	2.59 (−3.52, 8.68)	*P* = 0.404	0.61 (−6.04, 7.24)	*P* = 0.856
**Months from the baseline per 3 months** (Baseline = Ref.)	− 0.08 (−1.77, 1.61)	*P* = 0.929	0.07 (−1.98, 2.11)	*P* = 0.948
**Living situation** (Living with others = 1, Living alone = Ref.)	−9.42 (− 19.52, − 2.96)	*P* = 0.010	−9.72 (− 17.36, − 2.11)	*P* = 0.012
**Interaction term between months from the baseline and living with others**	1.81 (−0.23, 3.87)	*P* = 0.082	2.33 (− 0.08, 4.76)	*P* = 0.060
**Economic satisfaction** (not = 4, somewhat = 3, neither = 2, sufficiently = 1, very = Ref.)			−0.39 (− 4.15, 1.34)	*P* = 0.319
**Depression** (yes = 1, no = Ref.)			−5.35 (−9.26, −1.43)	*P* = 0.007
**MCI** (yes = 1, no = Ref.)			−3.20 (−9.92, 3.52)	*P* = 0.060
AIC	6547.6		5548.4	
BIC	6593.7		5606.1	
Log Likelihood	− 3263.8		− 2761.2	

All covariate represents values collected at baseline. *95%CI* 95% Confidence interval *Ref.* Reference

For assessing the effect of social frailty, each item in the KYCL were integrated into the model and estimates were calculated. The results are shown in Table 4. The results show that the mean LSA score of older adults who lived with others was significantly lower (β = − 8.76, 95%CI = − 18.52 to − 1.87, p = 0.012) than that of older adults who lived alone (Model 3). In addition, LSA scores were significantly lower among elderly people who spent most of the day inside the house rather than going outside (β = − 7.29, 95%CI = − 11.29 to − 3.29, *p* < 0.001), those who had hobbies (β = − 4.00, 95%CI = − 7.90 to − 0.11, *p* = 0.043); those who enjoyed talking to their neighbors (β = − 4.84, 95%CI = − 9.00 to − 0.69, *p* = 0.020); those who frequently went outside for work, shopping, walking, or hospital visits (β = − 5.70, 95%CI = − 9.90 to − 1.52, *p* = 0.007); those who trip and slip well in the house (β = − 4.85, 95%CI = − 8.77 to − 0.93, *p* = 0.015); and those who were unable to walk continuously for about 1 km (β = − 6.74, 95%CI = − 10.86 to − 2.60, *p* = 0.001). Estimates were the highest regarding living situation (β = − 8.76, 95%CI = − 18.52 to − 1.87, *p* = 0.012). Intercept of the LSA score was 84.31 points (95%CI = 75.27 to 92.84, *p* < 0.001).

**Table 4:** The associations of frailty and life-space mobility

	Model 3	*p*-value	Model 4	*p*-value
	Mean difference of LSA score by 1-unit increase of each explanatory variable		Mean difference of LSA score by 1-unit increase of each explanatory variable	
	(95%CI)		(95%CI)	
(Intercept)	84.31 (75.27, 92.84)	*P* < 0.001	86.78 (76.52, 96.95)	*P* < 0.001
**Age** (years old)	−0.38 (− 0.69, 4.02)	*P* = 0.020	− 0.28 (− 0.63, 0.06)	*P* = 0.108
**Female sex** (men = Ref.)	− 1.26 (− 6.50, 4.02)	*P* = 0.637	−2.82 (− 8.65, 3.05)	*P* = 0.342
**Months from the baseline per 3 months** (baseline = Ref.)	0.22 (− 1.58, 2.02)	*P* = 0.808	0.19 (− 1.87, 2.27)	*P* = 0.856
**Living situation** (Living with others = 1, living alone = Ref.)	−8.76 (− 18.52, − 1.87)	*P* = 0.012	− 7.42 (− 18.30, − 0.15)	*P* = 0.048
**Interaction term between months from** **the baseline and living with others**	1.44 (− 0.74, 3.63)	*P* = 0.195	1.81 (− 0.64, 4.27)	*P* = 0.147
**Frailty (each item of KYCL)**				
***Houseboundedness***				
spend most of time in house all day (yes = 1, no = Ref.)	−7.29 (− 11.29, − 3.29)	*P* < 0.001	− 7.03 (− 11.18, − 2.88)	*P* < 0.001
have hobbies and fun (no = 1, yes = Ref.)	−4.00 (− 7.90, − 0.11)	*P* = 0.043	−3.69 (− 7.79, 0.41)	*P* = 0.077
have neighbors you enjoy talking with (no = 1, yes = Ref.)	−4.84 (− 9.00, − 0.69)	*P* = 0.020	−5.21 (− 9.68, − 0.75)	*P* = 0.020
have anyone to visit each other (no = 1, yes = Ref.)	−2.47 (− 6.81, 1.87)	*P* = 0.263	−1.55 (− 6.10, 2.98)	*P* = 0.499
frequency of going out (less than 1 time/week = 1, 1 time/2 ~ 3 day = Ref.)	− 5.70 (− 9.90, − 1.52)	*P* = 0.007	−4.57 (− 9.00, − 0.14)	*P* = 0.042
***Falling***				
history of falling within a year (yes = 1, no = Ref.)	− 1.39 (− 4.90, 2.12)	*P* = 0.435	−2.10 (− 5.85, 1.65)	*P* = 0.269
fear of falling (yes = 1, no = Ref.)	− 1.43 (− 5.74, 2.87)	*P* = 0.513	−2.47 (− 7.08, 2.15)	*P* = 0.294
trip and slip in the house (yes = 1, no = Ref.)	− 4.85 (− 8.77, − 0.93)	*P* = 0.015	−3.99 (− 8.21, 0.23)	*P* = 0.063
can walk continuously for about 1 km (can’t = 1, can = Ref.)	−6.74 (− 10.86, − 2.60)	*P* = 0.001	− 6.62 (− 11.01, − 2.23)	*P* = 0.003
low vision (yes = 1, no = Ref.)	− 0.72 (− 5.59, 4.15)	*P* = 0.770	−1.69 (− 6.86, 3.47)	*P* = 0.517
***Poor nutrition***				
history of hospitalization within a year (yes = 1, no = Ref.)	− 0.99 (− 5.05, 3.07)	*P* = 0.631	−1.29 (− 5.56, 2.98)	*P* = 0.552
bad appetite (yes = 1, no = Ref.)	2.74 (− 2.03, 7.50)	*P* = 0.259	3.07 (− 1.93, 8.08)	*P* = 0.227
weight loss more than 3 kg (yes = 1, no = Ref.)	− 1.83 (− 6.78, 3.13)	*P* = 0.466	−1.79 (− 7.03, 3.45)	*P* = 0.499
a loss of more muscle and fat (yes = 1, no = Ref.)	0.73 (− 2.62, 4.09)	*P* = 0.667	0.76 (− 2.85, 4.38)	*P* = 0.677
amount of foodstuff that can be chewed (can’t chew much = 1, can chew well = Ref.)	− 2.41 (− 8.10, 3.28)	*P* = 0.406	−2.39 (− 8.28, 3.49)	*P* = 0.424
**Economic satisfaction** (not = 4, somewhat = 3, neither = 2, sufficiently = 1, very = Ref.)			− 0.37 (− 2.78, 2.03)	*P* = 0.207
**Depression** (yes = 1, no = Ref.)			−1.34 (− 5.49, 2.81)	*P* = 0.527
**MCI** (yes = 1, no = Ref.)			− 3.66 (− 9.36, 2.05)	*P* = 0.207
AIC	5815.5		5085.3	
BIC	5927.8		5207.3	
Log Likelihood	− 2882.7		− 2514.7	

All covariate represents values collected at baseline

*95%CI* 95% Confidence interval, *Ref.* Reference

After further adjusting for economic satisfaction, depression, and MCI in Model 4, the associations remained statistically significant. The mean LSA score of older adults who lived with others was significantly lower (β = − 7.42, 95%CI = − 18.30 to − 0.15, *p* = 0.048) than that of older adults who lived alone. The results show that the LSA scores were significantly higher in elderly individuals who spent most of the day inside the house (β = − 7.03, 95%CI = − 11.18 to − 2.88, *p* < 0.001); those who enjoyed talking to neighbors (β = − 5.21, 95%CI = − 9.68 to − 0.75, *p* = 0.020); those who frequently went outside for work, shopping, walking, or hospital visits (β = − 4.57, 95%CI = − 9.00 to − 0.14, *p* = 0.042); and those who were unable to walk continuously for about 1 km(β = − 6.62, 95%CI = − 11.01 to − 2.23, *p* = 0.003). Intercept of LSA score was 86.78 points (95%CI = 76.52 to 96.95, *p* < 0.001).

A comparison of baseline disease history is shown in Additional file 3: Appendix 1, physical characteristics in Additional file 3: Appendix 2, and psychological characteristics in Additional file 3: Appendix 3.

## Discussion

In this study, we found that community-dwelling elderly persons who lived with others had a smaller life-space at baseline (7.42 points) than those who lived alone, even after adjusting for the effects of age, gender, frailty, economic satisfactory, depression, and MCI. However, the timewise changes in life-space mobility of elderly adults who live alone were better than those who live with others. To date, living alone has been noted as a risk factor for social frailty [15, 16, 18]; however, it appears that those who live with others have another risk. One of the reasons for the diminished life-space mobility of elderly persons who live with others could be that they rely on others in daily life activities, such as for shopping, so their life-space mobility decreases. There is evidence suggesting that, when living with family, the lives of older adults can be extended by being support, providers rather than receivers alone, which would clearly support the old adage “it is a greater blessing to give than to receive.” Older adults wanting to extend their lives can be encouraged to provide more help to their families [42]. In addition, if they have no friends, elderly individuals who cannot live alone because of disease or handicap typically enter a geriatric health facility, such as a nursing home. Some elderly people do not wish to enter a nursing home after their partner dies; rather, they prefer to live alone.

In Japan, elderly people living with others are limited to use home help services because of support person’s presence. Under the long-term care insurance system currently in place in Japan, elderly people who live alone and are certified as needing long-term care are entitled to receive escort services provided by health workers when they go shopping and other activities. Conversely, even if they are certified as needing nursing care, elderly people who lives with others (including their elderly spouse) are restricted from receiving the same services. This could be said to be influenced by the specific cultural background of Japan, such as the prevention of confinement for elderly people who live alone. This study revealed that older people living with others have low life-space mobility and, thus, may require long-term care support. A recent study found that older women living alone are not always at risk for health issues, while adverse health outcomes among older adults living alone may be confounded by poor social networks [43]. In addition, there is evidence that elderly people living alone experience greater isolation from family members and emotional loneliness, compared to those living with their family, but are not necessarily highly isolated from their friends, may not feel socially isolated, and are more likely to participate in social activities on a regular basis [2].

Environmental and personal contextual aspects of life-space mobility have been previously addressed in a systematic review [44]. Environmental aspects include physical characteristics of the environment, interpersonal relationships, legislation, and use of products and technology. Personal contextual aspects include economic conditions, life adversities, employability, attendance at medical appointments or hospitalization, gender, race, age, lifestyle, different ways of facing problems, social background, and level of education.

In this study, there was no association between life-space mobility and age, gender, and history of hospitalization in Model 4. Regarding the coefficient of age on life-space mobility, in Model 4 we adjusted for possible intermediate variables including economic satisfaction, depression, and MCI, which could have offset the association of age with life-space mobility. This could be considered as over-adjustment bias due to an intermediate variable (or a descending proxy for an intermediate variable) on a causal path from exposure to outcome [45]. In fact, in Model 3, without these variables, there was a significant association between age and life-space mobility. Similarly, the regression coefficients of the adjustment variables require cautious interpretation. Gender has been noted as related to social frailty in various studies; social frailty may be more prevalent in women [16]. However, changes in life-space mobility were consistently unaffected by gender in this study. This point could not be clarified by the present data, thus, this should be studied in other future studies. No significant association of history of hospitalization with life-space mobility could have been owed to the fact that participants’ hospitalization history was not precise enough. We asked participants about their hospitalization history within the previous year, which means that the information varied from just 1 week ago to 1 year earlier.

Although prevention of social frailty is as important as preventing physical frailty or sarcopenia and decreased mental functioning, it may be more difficult to provide interventions that address social frailty. Health education or policy strategies to minimize social frailty do not guarantee success; more research is needed into intervention strategies to prevent social frailty. Another reason why elderly people who live with others have less life-space mobility is related to age and fear of falling [42]. However, in our study population, there was no association between life-space mobility and fear of falling, or history of falling within the past year. The reasons why we considered there were also fear of falling or history of falling as the intermediate variables between life-space mobility and houseboundedness. People with a tendency to stay indoors all day could have a lower history of falls because they do not go out. In addition, people might stay indoors because they fear falling. In this study, we focused on the effects of houseboundedness as an aspect of social frailty. Therefore, the effects of falling were treated as mediator variables. In fact, in the model that omitted houseboundedness variables, there was a significant association between history of falling or fear of falling and life-space mobility (see Supplemental Table 2). However, in addition to living situation, a shut-in tendency (remaining in the house), neighborhood friends, the frequency of going out, and the ability to walk continuously for a distance of 1 km were associated with LSA score. This result is consistent with a previous study where life-space mobility scores were associated with a person’s physical capacity and other factors that may limit mobility [46]. In addition, a previous study found that a sense of autonomy for physical performance explained more than 30% of the variation in life-space mobility [30], which supports the finding that perceptions of being able to walk more than 1 km continuously were affected in this study. Further studies are needed to explain mobility deficits and to plan appropriate interventions to address said deficits.

In addition, a systematic review that examined the association between life-space mobility and cognitive function in older adults concluded that a moderately strong relationship exists between life-space mobility and cognition, whether adjusted or unadjusted for covariates like socio-demographics, mental health, functional capacity, and comorbidities [47]. However, in this study, such associations were not found; however, a previous study’s results, which showed that the relationship between change in cognitive function and life-space mobility in older adults was not well-defined over an observation period of 2 years, [48] are consistent with our study.

Japan has been providing more generous care services to elderly individuals living alone. However, this study revealed that reduced living space among older individuals who live with their families has become a problem. Therefore, it is necessary to rethink the target of service provision.

## Conclusion

Using a multilevel growth model, we found that elderly persons who lived with others had significantly lower life-space mobility than those who lived alone. This suggests the need to further consider social frailty among elderly adults who live with others.

### Limitations

This study is limited in its generalizability because it included individuals who indicated their willingness to participate in the day service center’s care prevention campaign and who understood the purpose of the study and indicated their consent. In addition, 69.2% of the participants, despite having a low MoCA-J score and being below the cut-off point for MCI, were considered able to answer the questionnaire relatively correctly, in contrast to people who met the exclusion criteria. A previous study reported that 66% of the population-based survey participants (34% Caucasian, 52% African American, 11% Hispanic, and 2% other) [49], and 82.6% among community-dwelling older adults in Japan were judged below the cut-off of 26 points for detecting MCI [50]. As reported in these previous studies, cultural context and demographic factors need to be taken into consideration when applying the cut-off point.

## Supplementary Information

**Additional file 1:** **Supplemental Table 1.** EMMs of life-space mobility obtained by linear mixed models.

**Additional file 2:** **Supplemental Table 2.** The associations of falling and life-space mobility.

**Additional file 3.**

## Data Availability

The datasets generated during the current study are available from the corresponding author on request due to privacy/ethical restrictions.

## Abbreviations

LSA: life-space assessment
MCI: mild cognitive impairment
MoCA-J: Cognitive Assessment Instrument
GDS: Geriatric Depression Scale
KYCL: Kaigo-Yobo checklist
MAR: mission-at-random
