# Supplementary material for: Assessing timewise changes over 15 months in life-space mobility among community-dwelling elderly persons
Source: BMC Geriatr. 2020 Nov 25;20:502. doi: 10.1186/s12877-020-01882-4 (PMC7687835; doi:10.1186/s12877-020-01882-4)
Supplement: Supplementary file 3 — Additional file 3. [file 12877_2020_1882_MOESM3_ESM.docx]

**Appendix 1. Comparison of disease history between groups.**

**Living alone Living with others p-value**

Parkinson’s disease + Male 1(5.6%) 11(12.6%)

Female 0(0.0%) 5(5.9%) 1.000

Cerebral Hemorrhage+ Male 0(0.0%) 13(14.9%)

Female 2(3.3%) 5(5.9%) 0.111

Cerebral infarction+ Male 6(33.3%) 25(28.7%)

Female 2(3.3%) 19(22.4%) 0.450

High blood pressure+ Male 8(44.4%) 40(46.0%)

Female 32(52.2%) 35(41.2%) <0.001

Angina+ Male 2(11.1%) 11(12.6%)

Female 9(14.8%) 7(8.2%) 0.052

Myocardial infarction+ Male 2(11.1%) 7(8.0%)

Female 2(3.3%) 5(5.9%) 1.000

Gout+ Male 3(16.7%) 6(6.9%)

Female 2(3.2%) 2(2.4%) 1.000

Rheumatism+ Male 0(0.0%) 3(3.4%)

Female 1(1.6%) 6(7.1%) 1.000

Osteoporosis+ Male 1(5.6%) 7(8.0%)

Female 28(45.9%) 25(29.4%) 0.055

Fracture+ Male 4(22.2%) 22(25.3%)

Female 24(39.3%) 18(21.2%) <0.001

Arthropathy+ Male 1(5.6%) 8(9.2%)

Female 10(16.7%) 20(23.5%) 0.399

Backache+ Male 7(38.9%) 34(39.1%)

Female 32(52.5%) 38(44.7%) <0.01

Eye disease+ Male 7(38.9%) 36(41.4%)

Female 35(57.4%) 40(47.1%) <0.01

Dementia+ Male 1(5.6%) 7(8.0%)

Female 4(6.6%) 3(3.5%) 0.119

Diabetes+ Male 4(22.2%) 19(21.8%)

Female 6(9.8%) 11(12.9%) 0.274

Cancer+ Male 3(16.7%) 19(21.8%)

Female 6(9.8%) 11(12.9%) 0.142

**Appendix 2. Comparison of baseline physical characteristics between groups.**

**Living alone Living with others p-value**

**(N=75; 32.2%) (N=158; 67.8%)**

**Male (N=100; 42.9 %)**

Age (years old) 75.31±8.86 77.06±7.59 0.522

Height(cm) 163.84±7.89 164.23±6.44 0.867

Weight(kg) 62.50±10.50 62.75±9.57 0.937

Handgrip-right(kg) 27.11±11.87 26.12±8.26 0.773

Handgrip-left(kg) 21.71±8.67 25.31±7.98 0.175

Calf_right(cm) 34.06±3.93 34.46±3.27 0.735

Calf_left(cm) 34.04±3.63 34.48±3.20 0.682

Gait speed(sec) 11.01±13.80 6.47±3.02 0.242

Pain(mm) 36.86±33.66 23.30±27.69 0.180

**Female (N=133; 57.1%)**

Age (years old) 82.66±6.54 79.20±7.23 ＜0.05

Height(cm) 148.94±4.92 149.57±6.37 0.610

Weight(kg) 50.16±8.04 51.40±10.07 0.529

Handgrip-right(kg) 15.64±4.92 15.46±5.07 0.873

Handgrip-left(kg) 14.44±4.65 14.30±5.00 0.889

Calf_right(cm) 32.15±3.08 32.82±3.67 0.356

Calf_left(cm) 32.24±2.88 33.09±4.07 0.260

Gait speed(sec) 5.84±2.78 6.57±3.20 0.262

Pain(mm) 28.05±29.28 41.23±33.07 0.055

**Appendix 3. Comparison of baseline psychological characteristics between groups.**

**Living alone Living with others p-value**

**(N=75; 32.2%) (N=158; 67.8%)**

**Male (N=100; 42.9 %)**

Extraversion 7.72±2.16 7.86±2.42 0.814

Agreeableness 10.61±2.03 10.90±1.82 0.588

Conscientiousness 8.83±2.50 8.98±2.27 0.825

Neuroticism 7.88±2.26 6.97±2.30 0.141

Openness 8.39±1.75 7.80±2.46 0.242

**Female (N=133; 57.1%)**

Extraversion 8.45±2.12 8.13±2.32 0.405

Agreeableness 11.04±1.85 10.90±1.91 0.588

Conscientiousness 9.32±2.13 8.64±2.32 0.086

Neuroticism 7.36±2.19 7.82±2.32 0.249

Openness 7.71±1.73 7.07±2.52 0.082
